# The Influenza Virus Protein PB1-F2 Interacts with IKKβ and Modulates NF-κB Signalling

**DOI:** 10.1371/journal.pone.0063852

**Published:** 2013-05-21

**Authors:** Ana Luísa Reis, John W. McCauley

**Affiliations:** Division of Virology, MRC National Institute for Medical Research, London, United Kingdom; University of Edinburgh, United Kingdom

## Abstract

PB1-F2, a protein encoded by a second open reading frame of the influenza virus RNA segment 2, has emerged as a modulator of lung inflammatory responses but the molecular mechanisms underlying this are only poorly understood. Here we show that PB1-F2 inhibits the activation of NF-κB dependent signalling pathways in luciferase reporter assays. PB1-F2 proteins from four different viruses interact with IKKβ in yeast two-hybrid assays and by co-immunoprecipitation. PB1-F2 expression did not inhibit IKKβ kinase activity or NF-κB translocation into the nucleus, but NF-κB binding to DNA was severely impaired in PB1-F2 transfected cells as assessed by Electrophoretic Mobility Shift Assay. Neither the N-terminal 57 amino acid truncated forms nor the C-terminus of PB1-F2 were able to inhibit NF-κB dependent signalling, indicating that the full length protein is necessary for the inhibition.

## Introduction

Influenza A viruses have a significant impact on the human population as demonstrated not only by the mortality associated with infection, but also by their economic impact on health care systems around the world. In addition to seasonal epidemics, influenza A viruses have the potential to cause pandemics due to the circulation of novel subtypes against which the human hosts have little or no previous immunity. Such viruses are derived either by reassortment between human and animal influenza viruses, or the zoonotic transfer of viruses from animals [1].

The mechanisms of influenza pathogenesis involve an intricate interplay between virus virulence factors and host immune responses [1], [2]. Inflammation is an essential component of the innate immune response controlling early viral replication [3]. However, excessive production of proinflammatory cytokines and infiltration of neutrophils and macrophages can impair lung function, ultimately leading to death of the host, such that inflammatory response has emerged as critical in the development of lung pathology during infection with highly virulent 1918 H1N1 and H5N1 viruses [4]–[7].

The nuclear factor-κB (NF-κB) family of transcription factors has long been recognised as a key regulator of the inflammatory response [8]. A p65-p50 heterodimer is the primary target of the canonical pathway, which is associated with the inhibitory protein IκBα. The canonical NF-κB pathway is triggered by the engagement of pattern recognition receptors (PRR) including toll like receptors (TLRs) and retinoic acid inducible gene (RIG-I), and pro-inflammatory cytokines such as tumor necrosis alpha (TNFα) and interleukin-1 (IL-1). The recruitment of a set of adaptor proteins such as TIR-domain-containing adapter-inducing interferon-β (TRIF), myeloid differentiation primary response gene 88 (Myd88) and mitochondrial antiviral signaling protein (MAVS) culminates with the activation of the IκB kinase (IKK) complex and phosphorylation of IκBα, which results in its degradation and translocation of NF-κB dimers to the nucleus. Genes activated by NF-κB encode cytokines, chemokines, adhesion molecules and inducible nitric oxide synthase (iNOS) [9].

Several influenza proteins have been associated with countering the host response to infection: NS1 which modulates the interferon response amongst other responses of the cell [10]; the viral polymerase that binds to MAVS and inhibits IFNβ production [11], [12]; a polypeptide designated PB1-F2, encoded by a second open reading frame of RNA segment 2 [13]; and a recently discovered polypeptide encoded in a ribosomal frame-shifted reading frame in RNA segment 3, PA-X [14].

The role of NS1 has been the subject of extensive investigation [10] but the role of PB1-F2 is much less well understood and little is yet known about the action of PA-X. It is known that PB1-F2 expression from the 1918 H1N1 virus enhances the lung inflammatory response in an animal model of secondary bacterial infection characterized by infiltration of immune cells and a significant increase in the levels of pro-inflammatory cytokines [15]. This was also observed for other isolates, indicating that the pro-inflammatory properties are a general feature of different PB1-F2 proteins [16].

The molecular mechanisms that mediate the induction of hypercytokinemia and immune cell infiltration by PB1-F2 are poorly understood, but a variety of biochemical properties have been assigned to it. PB1-F2 contains a mitochondrial targeting sequence interacting with the permeability transition pore complex resulting in cytochrome C release and the loss of the mitochondrial membrane potential [13], [17], [18]. It can also form pores in lipid membranes [19], [20]. Monocytic cells have been shown to be highly susceptible to PB1-F2 induced apoptosis [13], [21] and this might be associated with the development of lung immunopathology [22]. However, the pro-apoptotic properties of PB1-F2 are strain specific [16], [23]. The PB1-F2 polypeptide has been reported to increase virus polymerase activity as a result of the interaction between PB1 and PB1-F2 [24], but in both a strain- and cell-type specific manner [25]. PB1-F2 has also been linked to the interferon response. Mice infected with a virus encoding PB1-F2 with a N66S substitution exhibit a delayed type I IFN induction resulting in uncontrolled virus replication and a consequent increased inflammatory response [26]. A recent study showed that the deletion of PB1-F2 enhances IFNβ expression in epithelial cells [27]. The inhibition of type I IFN response by PB1-F2 was reported to occur at the level of MAVS [27]–[29] thereby impacting on the activation of interferon regulatory factor-3 (IRF-3) [27]. However, other authors found a significantly higher IFNβ expression in human respiratory epithelial cells infected with a wild type virus when compared to a PB1-F2 knockout virus. This increased IFN expression was dependent on the activation of NF-κB [30] and occurred during *in vivo* infection [31].

Given the limited understanding of the molecular mechanisms underlying the impact of PB1-F2 on the development of lung inflammatory response and the central role of NF-κB signalling pathways in the initiation and maintenance of inflammation, here we evaluated the consequences of expression of PB1-F2 proteins from different virus strains on cellular responses mediated by NF-κB. We show that PB1-F2 inhibits activation of the canonical NF-κB pathways in transfected cells. Importantly, PB1-F2 is able to interact with IKKβ, the key kinase for both PRR and pro-inflammatory cytokines signalling cascades, consistent with our observation that NF-κB binding to its cognate DNA is impaired in cells expressing PB1-F2.

## Materials and Methods

### Cells

African green monkey Vero cells (European Collection of Cell Cultures) were maintained in Dulbecco’s modified Eagle medium (DMEM) supplemented with 10% foetal bovine serum (FBS), 50 U ml^−1^ penicillin, 50 µg ml^−1^ streptomycin and 10 mM glutamine.

### Plasmids

The PB1-F2 open reading frames (ORFs) from A/Quail/Hong Kong/G1/97(H9N2) (Q/HK97), A/Puerto Rico/8/34(H1N1) (PR8), A/turkey/England/50-92/91(H5N1) (T/Eng) and A/turkey/Turkey/1/2005(H5N1) (T/Tur) were amplified from viral RNA using the OneStep RT-PCR Kit (Qiagen) and cloned into pcDNA4 (Invitrogen) in frame with the carboxy-terminal myc tag. Similarly, DNA fragments encoding amino-terminal (residues 1–57) and carboxy-terminal (residues 58-STOP) PB1-F2 peptides from the same viruses were amplified and cloned into pcDNA4.

The reporter plasmid for NF-κB [p(PRDII)5tkΔ(−39)lucter] [32] and expression vectors for TRIF, MAVS, IKKα and IKKβ were kindly provided by Prof. S. Goodbourn (St. George's, University of London); pcDNA3 harbouring the human p65 gene was a kind gift of Dr. S. Ley (NIMR); pUNO containing the human MyD88 ORF was from Invivogen and the pSV-β plasmid containing a β-galactosidase gene under control of the SV40 early promoter was from Promega.

### GST Recombinant Proteins

The p65 transcription activation domain (residues 354–551) was amplified by PCR using pCDNA3-p65 as a template, cloned into the pGEX4T-1 (GE Healthcare) plasmid and used to transform competent *Escherichia coli* strain BL21 (Stratagene); the expression of the recombinant p65 was induced with 1 mM IPTG for 2 hours. GST-p65 was purified using Glutathione Sepharose 4B (GE Healthcare) following the manufactureŕs instructions. GST- IκBα (residues 1–54) was kindly provided by Dr. K. Rittinger (NIMR).

### Luciferase Reporter Assay

Vero cells (1×10^5^ cells/well, in a 24 well plate) were co-transfected with 100 ng of the NF-κB reporter plasmid, 25 ng of pSV-β and 300 ng of one of the pcDNA4-PB1-F2 plasmids or the empty plasmid, according to the Lipofectamine 2000 protocol (Invitrogen). Forty eight hours post-transfection, the cells were stimulated with 25 µg ml^−1^ Poly (I:C) (Sigma), 20 ng ml^−1^ TNFα (Peprotech), 1 µg ml^−1^ LPS (Sigma) or 10 ng ml^−1^ IL-1β (Peprotech) for five hours. After stimulation, the cells were lysed with 0.1 M potassium phosphate pH 7.8, 1% Triton X-100, 1 mM DTT, 2 mM EDTA, and the luciferase activity was measured using the luciferase assay system (Promega) according to the manufacturer`s protocol. Transfection efficiency was standardised against β-galactosidase activity measured using Galacton-Plus (Applied Biosystems).

In an alternative assay, cells were co-transfected with 100 ng of reporter plasmid, 25 ng of pSV-β, the indicated amounts of plasmids expressing the different components of the NF-κB pathway and 300 ng of the PB1-F2 constructs. Forty eight hours post transfection, the luciferase and galactosidase activities were measured as above.

### Yeast Two-hybrid Assay

Q/HK97 PB1-F2 ORF was excised from the pcDNA4 expression vector and subcloned into the pGADT7 and pGBKT7 plasmids in frame with the GAL4 activation domain (AD) and GAL4 binding domain (BD) respectively. Similarly, IKKα and IKKβ were subcloned into the pGADT7 plasmid in frame with the GAL4 activation domain.

*Saccharomyces cerevisiae* strain AH109 cells were co-transformed with different combinations of pGADT7 and pGBKT7 constructs, using lithium acetate as described in the Clontech Yeastmaker™ Yeast Transformation System 2 user manual. Double transformants were selected by growth at 30°C on synthetic dropout (SD) medium lacking leucine and tryptophan (SD/−Leu/−Trp) and then streaked on SD/-Leu/-Trp/-His +10 mM 3-Amino-1,2,4-triazole (3-AT) plates. The colonies surviving this selection were replica plated on medium containing 40 µg ml^−1^ X-α-gal (Clontech).

### Immunoprecipitation

Vero cells (2.4×10^6^ cells, 100 mm plate) were transfected with 12 µg of one of the pcDNA4-PB1-F2 plasmids or the empty plasmid and 12 µg of Flag tagged pcDNA3-IKKβ. Forty-eight hours post-transfection, the cells were washed with phosphate buffered saline (PBS) and lysed with 500 µl of cell lysis buffer (Cell Signaling Technology) supplemented with a protease inhibitor cocktail (Sigma). Cellular extracts were centrifuged for 10 min at 13000×*g* and supernatants were incubated overnight at 4°C with protein G Dynabeads (Invitrogen) previously coated with monoclonal mouse anti-myc antibody (Sigma, M4439). The Dynabeads were then washed 4 times with PBS +0.02% Tween 20 and finally resuspended in 1× SDS loading buffer (0.06 M Tris-HCl pH 6.8, 1.7% SDS, 6% glycerol, 0.8% β-mercaptoethanol, 0.002% bromophenol blue). After incubation at 95°C for 10 min, supernatants were resolved by sodium dodecyl sulfate - polyacrylamide gel electrophoresis (SDS-PAGE) and proteins were transferred to a polyvinylidene difluoride (PVDF) membrane. The immunoblots were blocked for two hours at room temperature with Tris Buffered Saline pH 7.4 (TBS, Fisher Scientific) +0.1% Tween 20 containing 5% non-fat milk and incubated overnight at 4°C with monoclonal mouse anti-myc and rabbit anti-Flag (Cell Signaling Technology, 2368) both diluted 1∶1000 in TBS +0.1% Tween 20 containing 5% bovine serum albumin (BSA). After washing, the membrane was probed with goat anti-rabbit Dylight 800 (Thermo Scientific, 35571) and goat anti-mouse Alexa Fluor 680 (Invitrogen, A21058) diluted 1∶2000 and analysed on the Odyssey Infrared Imaging System (LI-COR).

### Immunoblotting

Vero cells (5×10^5^ cells/well, 6 well plate) were transfected with 3 µg of one of the pcDNA4-PB1-F2 plasmids or the empty plasmid. Forty-eight hours post-transfection, the cells were washed with PBS and lysed directly in 100 µl 1×SDS loading buffer. Lysates were run on a SDS-PAGE gel, transferred to a PVDF membrane and non-specific binding was blocked as above. The membranes were incubated overnight at 4°C with monoclonal mouse anti-myc antibody diluted 1∶1000 in TBS+0.1% Tween 20+5% BSA. After washing, the membrane was probed with goat anti-mouse Alexa Fluor 680 diluted 1∶2000 and analysed as described for immunoprecipitation.

### Immunofluorescence

Vero cells (1×10^5^) on glass coverslips were transfected with 300 ng of one of the pcDNA4-PB1-F2 plasmids or the empty plasmid and 300 ng of pcDNA3-IKKβ. Forty-eight hours post-transfection, the cells were fixed with 4% formaldehyde, permeabilised with PBS +0.2% Triton X-100 and blocked with PBS +1% BSA. The samples were then incubated for 1 hour with monoclonal mouse anti-myc antibody diluted 1∶100 and rabbit anti-Flag antibody, diluted 1∶500 in blocking buffer, washed with PBS, and incubated for 1 hour with anti-mouse Alexa Fluor 594 and anti-rabbit Alexa Fluor 488 secondary antibodies (Invitrogen) diluted 1∶500 in blocking buffer, all at room temperature. The coverslips were mounted in ProLong antifade medium (Invitrogen) and examined using a confocal microscope (Leica DMIRE2).

For assessment of p65 nuclear translocation, Vero cells were transfected with 400 ng of pcDNA4-PB1-F2Q/HK97 with Lipofectamine 2000. Forty-eight hours post-transfection, the cells were stimulated with 1 µg ml^−1^ LPS+800 ng ml^−1^ CD14 (R&D Systems) for 1 hour. Cells were then washed with PBS and fixed with 4% formaldehyde for 20 minutes. After permeabilisation, the cells were blocked and incubated for 1 hour with monoclonal mouse anti-myc antibody diluted 1∶100 and rabbit anti-p65 antibody (Santa Cruz Biotechnology, SC-372) diluted 1∶50. Coverslips were then washed with PBS, incubated with secondary antibodies and mounted as described above.

### Kinase Assay

Vero cells were transfected with pcDNA4-PB1-F2Q/HK97 or empty plasmid and pcDNA3-IKKβ as described for immunoprecipitation. Extracts were prepared using cell lysis buffer and IKKβ was immunoprecipitated by incubating the lysates for 2 hours at 4°C with protein G Dynabeads coated with rabbit anti-Flag antibody. After extensive washing with cell lysis buffer and kinase buffer (20 mM Hepes pH 7.5, 10 mM MgCl_2_, 2 mM DTT, 1 mM Na_3_VO_4_, 2 mM NaF, 20 mM β-glycerophosphate, supplemented with protease inhibitor cocktail), the beads were resuspended in 20 µl kinase buffer containing 1 µM ATP and 2 µg of GST-IκBα or GST-p65. Finally, 0.0925 MBq ^32^P ATP (originally 111 TBq/mmol) was added to each tube and the mix was incubated at 30°C for 5, 10 or 20 min. The reactions were stopped by adding 4 µl 6× SDS loading buffer and incubating at 95°C for 10 min. Samples were separated by SDS-PAGE and the phosphorylation of the substrates was assessed by autoradiography.

### Electrophoretic Mobility Shift Assay (EMSA)

Vero cells expressing PB1-F2Q/HK97 were selected using the MACSelect™ K^k^ System (Miltenyi Biotec). Briefly, Vero cells (2.4×10^6^ cells, 100 mm plate) were transfected with 22.5 µg of pcDNA4-PB1-F2Q/HK97 or empty plasmid and 2.25 µg of the pMACS K^k^.II construct. Forty eight hours post-transfection, the cells were trypsinized and incubated with MACSelect microbeads coated with anti-K^k^ antibody. Cells bound to the microbeads were positively selected with the use of a magnetic field and 1×10^6^ cells were seeded per 60 mm plate. After adhering to the plate (5 hours), cells were treated with 1 µg ml^−1^ LPS +800 ng ml^−1^ CD14 for one hour. Next, the cells were washed with PBS and nuclear extracts were prepared by lysing the cells in 10 mM Hepes pH 7.5, 1.5 mM MgCl_2_, 10 mM KCl, 0.125% NP-40 and extracting nuclear proteins with 20 mM Hepes pH 7.5, 25% glycerol, 0.42 M NaCl, 1.5 mM MgCl_2_, 0.2 mM EDTA.

Protein-DNA complexes were formed by incubating 10 µg of nuclear extract for 20 minutes at room temperature with a NF-κB (AGTTGAGGGGACTTTCCCAGGC) 3′ end biotinylated probe. EMSA was performed with the LightShift Chemiluminescent EMSA Kit (Thermo Scientific) according to the manufacturer's protocol.

### Statistical Analysis

Statistical analysis was performed using GraphPad Prism6 software. Significant differences between groups were determined using one-way ANOVA followed by Dunnett's multiple comparison test.

## Results

### PB1-F2 Varies in its Ability to Modulate NF-κB Signalling

To evaluate the impact of PB1-F2 expression on NF-κB dependent signalling pathways, PB1-F2 ORFs from the prototype laboratory strain PR8, from a H9N2 virus isolated from quail (Q/HK97), and those from two H5N1 viruses, both from turkeys (T/Eng and T/Tur) but isolated 14 years apart and showing significant sequence diversity (Fig. S1) were cloned into a eukaryotic expression vector in frame with a myc tag. Each of the PB1-F2 constructs were transfected into a range of different cell lines. PB1-F2 was shown to be consistently detected by both Western blotting and immunofluorescence in Vero cells but inconsistently in other mammalian cell lines examined (data not shown), so Vero cells were chosen for this study.

**Figure 1:**
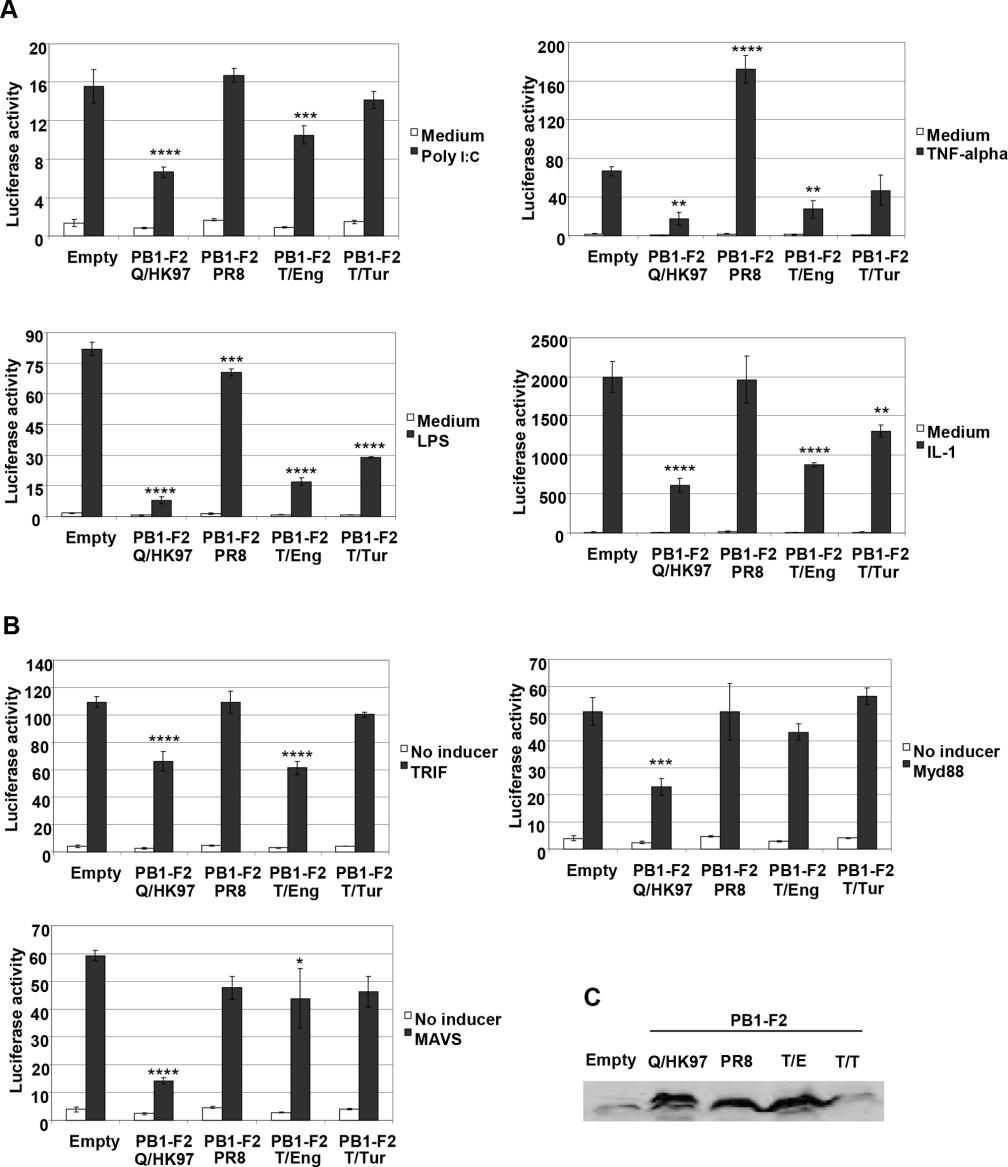
PB1-F2 modulates NF-κB signaling. A) Vero cells were co-transfected with the NF-κB reporter and either each of the different PB1-F2 expression vectors or the empty pcDNA4 plasmid along with the β-galactosidase control plasmid. Forty eight hours post-transfection, the cells were either induced or not induced with 25 µg ml^−1^ Poly (I:C), 20 ng ml^−1^ TNFα, 1 µg ml^−1^ LPS or 10 ng ml^−1^ IL-1β for five hours, cell extracts were made and the luciferase and galactosidase activites were measured. B) Vero cells were co-transfected with the NF-κB reporter and either each of the different PB1-F2 expression vectors or the empty pcDNA4 plasmid, the β-galactosidase control plasmid and 100 ng of TRIF, Myd88 or MAVS expressing vectors. Forty eight hours post-transfection, the cells were lysed and the luciferase and galactosidase activities were measured. Data are representative of at least 3 independent experiments. Bars represent average values and standard deviations of firefly luciferase activities from triplicate samples normalized to the expression of galactosidase. Asterisks indicate results significantly different from control empty vector (one-way ANOVA followed by Dunnett's test; *, P≤0.05; **, P≤0.01; ***, P≤0.001; ****, P≤0.0001). C) Vero cells in a 6 well plate, were transfected with 3 µg of each of the different PB1-F2 constructs or the empty pcDNA4 plasmid. Forty eight hours post-transfection, the cells were lysed and PB1-F2 expression was detected by immunoblotting using a monoclonal mouse anti-myc antibody.

A reporter plasmid containing a luciferase gene under the control of the PRDII element of the IFNβ promoter, responsive to NF-κB activation [32], was used to quantify the response of cells to both PRR and pro-inflammatory cytokine activated pathways (Fig. 1A). Luciferase activity was strongly induced after the stimulation of empty plasmid transfected cells with Poly I:C, LPS, TNFα and IL-1β. The induction of the reporter was reduced in cells transfected with PB1-F2 plasmids derived from the avian influenza viruses indicating that PB1-F2 is able to inhibit NF-κB mediated signalling. In contrast to the results for PB1-F2 from avian viruses, no significant decreases in luciferase activities were observed in cells expressing PB1-F2 from PR8, the human prototype virus. The inability of PB1-F2 from PR8 to inhibit NF-κB signalling cannot be ascribed to lower level of protein expression, since the latter was comparable to PB1-F2 from Q/HK97 and T/Eng (Fig. 1C). Therefore, our results indicate that PB1-F2 from different viruses can vary in its ability to modulate NF-κB in transfected cells.

To extend these results, the ability of PB1-F2 to modulate NF-κB activity induced by the overexpression of key components of signalling pathways initiated by PRR engagement was also examined (Fig. 1B). Overexpression of TRIF, Myd88 or MAVS increased luciferase activity when compared to control cells but was reduced when co-expressed with PB1-F2 from Q/HK97; co-expression of PB1-F2 from T/Eng caused a reduction of luciferase activity stimulated by TRIF, but no significant reduction in luciferase activity was seen with PB1-F2 from T/Tur. PB1-F2 from T/Tur showed lower levels of protein expression (Fig. 1C) compared with the levels seen for the other proteins, which may be associated with its failure to inhibit the induction of the reporter in the assay based on over-expression of signalling intermediates. As in the stimulation assays, no reductions were observed in cells co-expressing PB1-F2 from PR8.

Importantly, PB1-F2 showed no inhibition of signalling when reporters dependent on the activation of the transcription factors Signal Transducer and Activator of Transcription STAT1 and STAT2 (gamma activated site and interferon stimulated response element) were tested (Fig. S2), indicating that the effect seen was not due to a general impairment of cell signalling pathways.

### PB1-F2 Interacts with IKKβ

**Figure 2:**
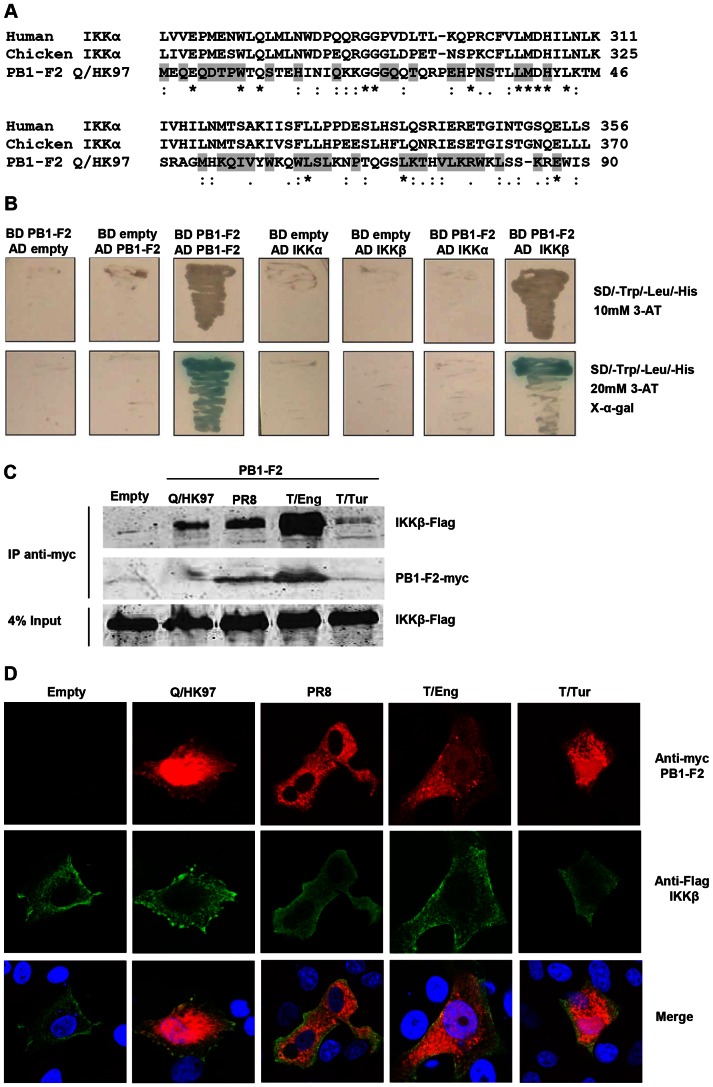
PB1-F2 has sequence similarity with IKKα and interacts with IKKβ. A) Alignment of PB1-F2 Q/HK97 protein sequence with human and chicken IKKα using ClustalW (*denotes identical residues, : denotes conservative substitutions and. denotes semi-conservative substitutions). The grey shading denotes the conserved residues in PB1-F2 from PR8, T/Eng and T/Tur. B) Yeast cells were independently transformed with different combinations of pGADT7 (AD) and pGBKT7 (BD) constructs and plated on selective medium. Yeast cells co-transformed with BD PB1-F2 and AD PB1-F2 were used as a positive control for protein-protein interaction. C) Vero cells were transfected with the indicated constructs and forty eight hours after transfection, protein complexes were immunoprecipitated from cellular lysates using a monoclonal anti-myc antibody. Proteins in the immunoprecipitates and in the whole cell lysates (4% input) were evaluated by Western blot using anti-Flag and anti-myc antibodies. D) Vero cells were transfected with 300 ng of the different PB1-F2 expressing vectors and 300 ng of pcDNA3-IKKβ. The cellular localisation of the proteins was analyzed by confocal microscopy using anti-myc (PB1-F2, red) and anti-Flag (IKKβ, green) antibodies.

Activation of the NF-κB canonical pathway relies on the activation of the IKK complex and consequent phosphorylation of IκB proteins and translocation of NF-κB molecules to the nucleus. Since several viral proteins have evolved sequence and structural homologies to host proteins in order to modify critical pathways of the immune response, we searched for sequence homology between PB1-F2 and IKKγ, IKKα, IKKβ, IκBα and the different members of the NF-κB family of transcription factors. The observation that PB1-F2 has some sequence similarity to both human and chicken IKKα (Fig. 2A) and that in mammalian cells IKKα and IKKβ form homo and heterodimers associated, or not, with IKKγ prompted us to test whether the PB1-F2 protein is able to directly interact with IKKα and/or IKKβ. First, a yeast two-hybrid assay was carried out. Yeast expression vectors with PB1-F2 from Q/HK97 fused to either the GAL4 AD or the GAL4 BD and IKKα/IKKβ fused to the GAL4 AD were produced and transformed into yeast cells in different combinations. Yeast cells co-transformed with AD-PB1-F2 plus BD-PB1-F2, used to show that the PB1-F2 constructs were able to dimerise [24], survived selection in medium lacking histidine and induced galactosidase activity (Fig. 2B). Similarly, the cells transformed with BD-PB1-F2 plus AD-IKKβ were able to grow in selective medium and express galactosidase, in contrast BD-PB1-F2 plus AD-IKKα did not grow. These results indicate that PB1-F2 interacted with IKKβ but not with IKKα.

The interaction between PB1-F2 and IKKβ in mammalian cells was then tested following transfection of expression plasmids and immunoprecipitation (Fig. 2C). To assess if the interaction with IKKβ is a general feature of PB1-F2, the proteins derived from the viruses PR8, T/Eng and T/Tur were also included in this experiment. Immunoprecipitation of PB1-F2 using an anti-myc tag antibody resulted in the co-precipitation of IKKβ with each PB1-F2 tested, suggesting that the different abilities to modulate NF-κB signalling pathways are not directly related to their ability to interact with IKKβ. Instead, the amount of co-precipitated kinase seemed to be proportional to the amount of PB1-F2 present in the precipitate. PB1-F2 was not detected by Western blot in the whole cell input (4%), indicating low levels of protein expression.

Confocal microscopy of cells transfected with the different PB1-F2 constructs and IKKβ showed areas of protein co-localisation (Fig. 2D), further supporting the results obtained in the yeast two hybrid and co-immunoprecipitation studies.

### PB1-F2 Inhibits NF-κB Activation Induced by IKKβ Overexpression

**Figure 3:**
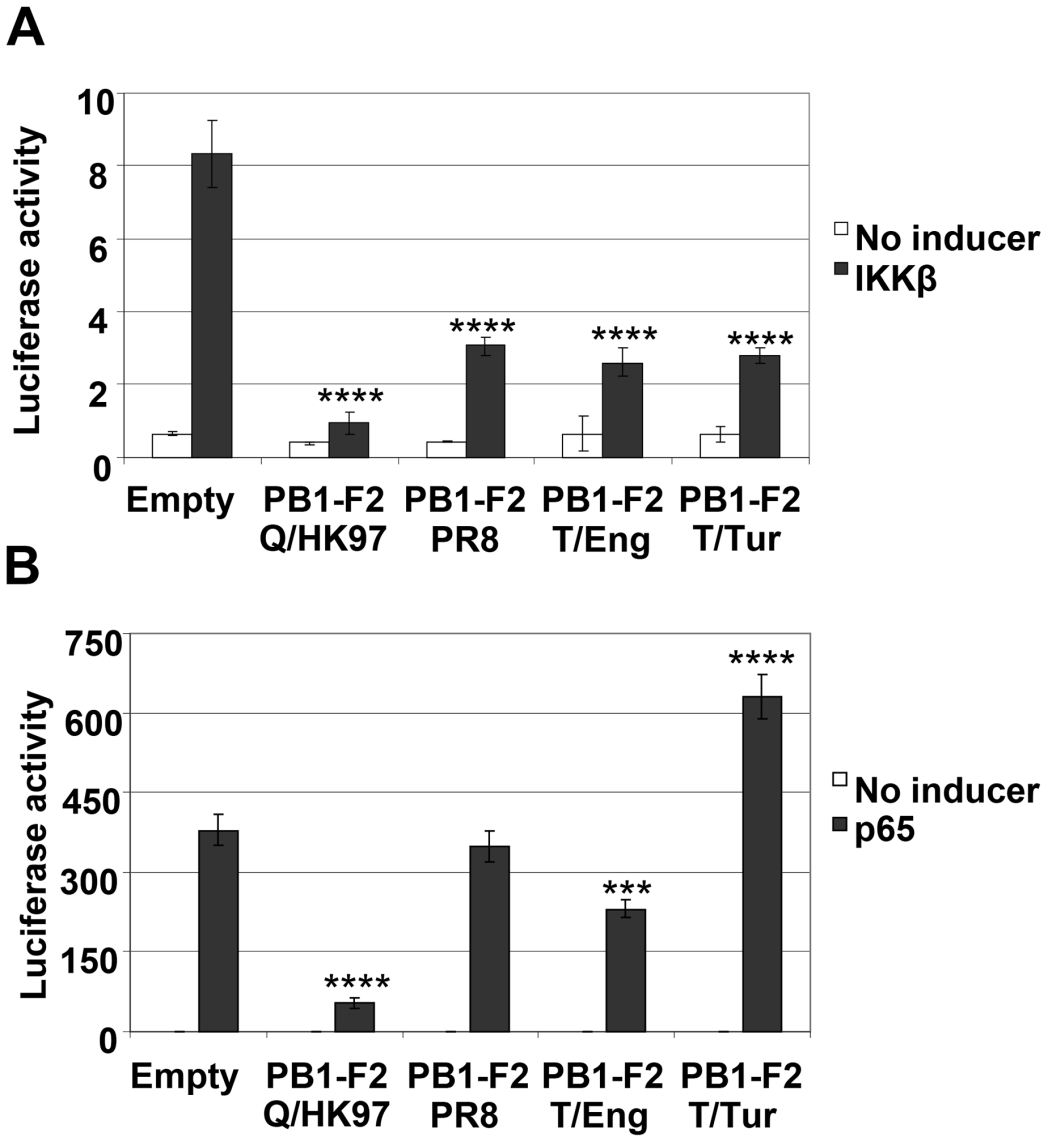
PB1-F2 proteins from different influenza isolates inhibit NF-κB activation induced by IKKβ overexpression. Vero cells were co-transfected with the NF-κB reporter and either each of the different PB1-F2 expression vectors or the empty pcDNA4 plasmid, the β-galactosidase control plasmid and 100 ng of pcDNA3-IKKβ (A) or 5 ng of pcDNA3-p65 (B). Forty eight hours post-transfection, the cells were lysed and the luciferase and galactosidase activities were measured. Data are representative of at least 3 independent experiments. Bars represent average values and standard deviations of firefly luciferase activities from triplicate samples normalized to the expression of galactosidase. Asterisks indicate results significantly different from control empty vector (one-way ANOVA followed by Dunnett's test; ***, P≤0.001; ****, P≤0.0001).

To investigate whether PB1-F2 interaction with IKKβ would result in impairment of NF-κB dependent transcription induced by IKKβ, cells co-transfected with IKKβ and the empty plasmid or the different PB1-F2 constructs were evaluated by luciferase assay (Fig. 3A). Overexpression of IKKβ resulted in robust activation of the PRDII reporter in cells transfected with the empty plasmid. However, in cells co-expressing the various PB1-F2s this activation was much lower, indicating that PB1-F2 proteins from each of the influenza viruses examined inhibit NF-κB activation induced by IKKβ overexpression. This result raises the possibility that the absence of an impact of PB1-F2 PR8 in stimulated cells (Fig. 1) could be a consequence of additional effects of this protein within these signalling pathways.

Having established that PB1-F2 inhibits the ability of IKKβ to induce NF-κB dependent transcription, the effect of PB1-F2 in cells overexpressing p65, the central transcription factor of the canonical NF-κB pathway, was evaluated (Fig. 3B). In cells transfected with the empty plasmid there was a strong induction of luciferase activity, which was significantly reduced in cells co-expressing PB1-F2 Q/HK97, while the other 3 PB1-F2 proteins induced varied effects.

### Full Length PB1-F2 is Necessary for Inhibition of NF-κB Dependent Signalling

**Figure 4:**
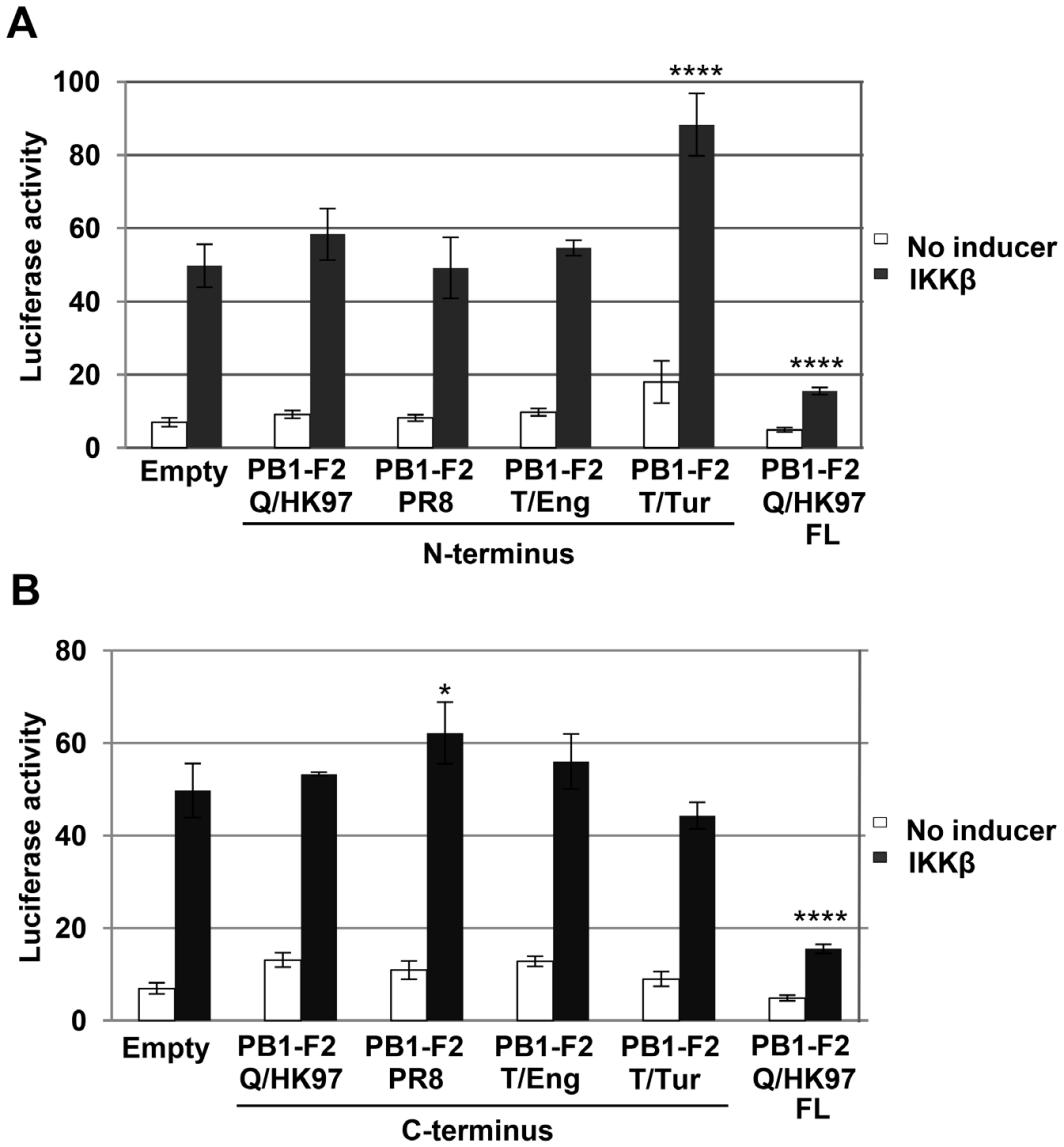
The full length PB1-F2 protein is necessary to inhibit NF-κB signaling. Vero cells were co-transfected with the NF-κB reporter, the different N-terminal (A) or C-terminal (B) PB1-F2 constructs or empty pcDNA4, the β-galactosidase control plasmid and 100 ng of pcDNA3-IKKβ. Cells transfected with the full length (FL) PB1-F2 Q/HK97 expression vector were used as a positive control for inhibition of NF-κB signalling. Forty eight hours post-transfection, the cells were lysed and the luciferase and galactosidase activities were measured. Data are representative of at least 3 independent experiments. Bars represent average values and standard deviations of firefly luciferase activities from triplicate samples normalized to the expression of galactosidase. Asterisks indicate results significantly different from control empty vector (one-way ANOVA followed by Dunnett's test; *, P≤0.05; ****, P≤0.0001).

Most avian isolates encode an intact PB1-F2, several swine isolates contain truncated forms of the protein (premature stops after 11, 25 and 34 codons), human seasonal A (H1N1) viruses isolated between 1950 and early 2009 carry a 57 amino acid truncated form [33] and the recent A(H1N1)pdm09 virus encodes only the first 11 amino acids of the protein. Therefore, we assessed whether an N-terminal truncated form of the protein (residues 1 to 57) or its C-terminal portion (residues 58 to the protein C terminus) are sufficient to inhibit NF-κB dependent signalling. The nucleotide sequences encoding these PB1-F2 fragments were cloned into the same expression vector as the full length protein and their expression in transfected cells was confirmed by immunofluorescence (Fig. S3). Subsequently, they were examined using the NF-κB luciferase reporter assay in cells overexpressing IKKβ. Cells transfected with the full length PB1-F2 proteins were tested in parallel for inhibition of NF-κB signalling (only PB1-F2 Q/HK97 is shown). Both the N-terminal portion (Fig. 4A) and C-portion (Fig. 4B) of PB1-F2 were unable to inhibit the induction of the reporter, implying that the full length protein is necessary to inhibit NF-κB dependent signalling.

### NF-κB Binding to DNA is Impaired in Cells Expressing PB1-F2

**Figure 5:**
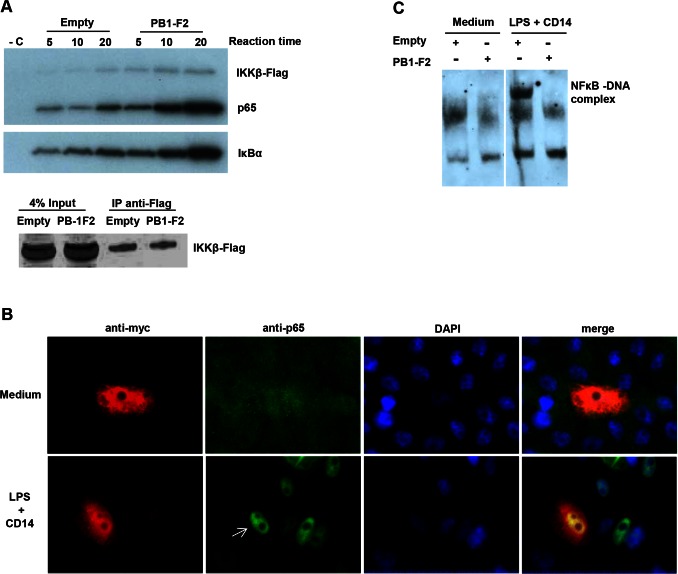
PB1-F2 increases IKKβ kinase activity but inhibits NF-κB binding to DNA. A) Vero cells were transfected with empty pcDNA4 or pcDNA4-PB1-F2Q/HK97 and pcDNA3-IKKβ. Forty eight hours post transfection, cells were lysed and IKKβ was immunoprecipitated using rabbit anti-Flag antibody. The kinase assay was performed for the indicated times in the presence of 0.0925 MBq ^32^P ATP, using GST-p65 and GST-IκBα as substrates. As a negative control (- C) non-transfected cells were used in the same experiment. Phosphorylation was detected by autoradiography. The levels of Flag-IKKβ present in the whole cell lysates (input) and immunoprecipitates (IP) were assessed by immunoblotting using rabbit anti-Flag antibody. B) Vero cells were transfected with pcDNA4-PB1-F2Q/HK97 and stimulated with 1 µg ml^−1^ LPS +800 ng ml^−1^ CD14 during one hour or left untreated (medium only). The cellular localisation of endogenous p65 was visualized using a rabbit anti-p65 antibody (green). Cells expressing PB1-F2 were identified (arrow) using a monoclonal mouse anti-myc antibody (red) and nuclei were stained with 4',6-diamidino-2-phenylindole (DAPI). C) Vero cells were transfected with empty pcDNA4 or pcDNA4-PB1-F2Q/HK97 and pMACS K^k^.II plasmid. Cells expressing K^k^ were selected using magnetic microbeads coated with anti-K^k^ antibody. These cells were stimulated with 1 µg ml^−1^ LPS +800 ng ml^−1^ CD14 for one hour or left untreated (medium only). Nuclear extracts were prepared and incubated with a NF-κB biotinylated probe. Complexes were resolved in TBE polyacrylamide gel, transferred to a membrane and biotinylated DNA was detected by chemiluminescence.

In addition to its role in IκBα phosphorylation, IKKβ phosphorylates p65 at Ser536 [34], [35], an event necessary for optimal transcriptional activity [35], and at Ser468 [36], which has been associated with p65 ubiquitination and subsequent degradation [37], [38]. To characterize better the molecular mechanism(s) employed by PB1-F2 to modulate IKKβ function, the catalytic activity of IKKβ was examined using an *in vitro* kinase assay in the presence or absence of PB1-F2 Q/HK97 with recombinant GST-IκBα and GST-p65 as substrates. IKKβ was immunoprecipitated from cells transfected with empty plasmid or the PB1-F2 construct and the phosphorylation status of IκBα and p65 was evaluated after addition of ^32^P ATP and incubation for different times (Fig. 5A). Surprisingly, IKKβ immunoprecipitated from cells expressing PB1-F2 showed increased catalytic activity, demonstrated by the enhancement of phosphorylation of both IκBα and p65. Furthermore, IKKβ self-phosphorylation was also increased. Importantly, the same amounts of IKKβ-Flag were detected in the immunoprecipitates from empty and PB1-F2 transfected cells (Fig. 5A), indicating that the observed differences did not result from different quantities of kinase present in the reaction.

Phosphorylation of IκBα results in its ubiquitination and degradation, thus releasing the inhibitory effects on p65 and favoring the nuclear localisation of the p65-p50 heterodimer. To test if PB1-F2 inhibits this fundamental step of NF-κB signalling, the cellular localisation of endogenous p65 was evaluated by immunofluorescence. In untreated cells, p65 was detected both in the cytoplasm and in the nucleus (Fig. 5B). As expected, stimulation of cells with LPS plus soluble CD14 [39] resulted in an obvious nuclear accumulation of p65. Importantly, the same increase in p65 nuclear staining was observed in cells expressing PB1-F2 Q/HK97, indicating that PB1-F2 does not inhibit the nuclear translocation of the p65-p50 heterodimer.

In the nucleus NF-κB dimers bind to promoters containing κB consensus sequences. The impact of PB1-F2 on NF-κB binding to its cognate DNA, analyzed by EMSA, was evaluated in Vero cells enriched for the expression of PB1-F2 using the MACSelect™ K^k^ System; cells transfected with the empty plasmid were treated in parallel. Cells were stimulated with LPS plus soluble CD14, nuclear extracts were prepared and incubated with a NF-κB probe. A shift of the probe was seen when extracts from cells transfected with the empty plasmid were used (Fig. 5C). This shift was not observed in cells expressing PB1-F2, indicating that binding of NF-κB transcription factors to DNA is impaired in these cells.

## Discussion

NF-κB signalling plays a central role in host defences against invading pathogens not only by initiating the innate immune response but also by orchestrating the development of acquired immunity. Being directly activated by PRR engagement, NF-κB induced genes are among the first to be transcribed in response to infection, and are a target for subversion of the host immunity by bacterial and viral pathogens. Several viral proteins have evolved to modify NF-κB signalling, impacting at different levels of its activation [40]; we submit that PB1-F2 might be included in this group of viral proteins, being able to modulate NF-κB activation and interact with IKKβ, the critical kinase of the canonical pathway.

PB1-F2 has a clear effect on virus pathogenesis that might correlate with an ability to alter NF-κB signalling. It was recently reported that infection of murine monocytic cells with an influenza virus lacking PB1-F2 (ΔPB1-F2) induced higher levels of IL-6 and IL-1β than with the wt virus [41]. Similar results have been obtained using ferret blood derived macrophages, where the absence of PB1-F2 resulted in significantly increased expression of IL-6, IL-1β and IL-8 [42] - all cytokines expressed under the control of NF-κB and potentially susceptible to modulation through PB1-F2.

PB1-F2 has been shown to influence the severity of secondary bacterial infection [15] and IFNγ has been shown to inhibit anti-bacterial defense during recovery from influenza virus infection [43]. These observations might be linked if PB1-F2 interacts with IKKβ since IKKβ suppresses IFNγ signalling in macrophages through inhibition of STAT1 activation [44]. Furthermore, a link between the effects of PB1-F2 on apoptosis in macrophages [13], [21] and IKKβ is also possible since activation of NF-κB signalling is critical for macrophage survival after TLR and TNFR1 engagement [45], [46].

Our finding that PB1-F2 is able to interact with IKKβ offers a possible target for the modulation of NF-κB signalling by this virus protein but the molecular mechanism of NF-κB inhibition by PB1-F2 is not fully characterized.

In our experiments full length PB1-F2 was necessary for inhibition of the NF-κB dependent pathways. To dissect the impact of PB1-F2 in infected cells and its role in NF-κB signalling, without other constrains such as N40 expression [47] or interaction with PB1 [24], generation of viruses with substitutions in PB1-F2 residues necessary for NF-κB inhibition and an examination of their properties *in vitro* and *in vivo* is required. However, we could not identify such residues, since single and multiple substitutions of amino acids in the region with highest similarity to IKKα did not alter the effect of PB1-F2 on NF-κB signalling in transfected cells (data not shown).

A recent study mapped the pro-inflammatory domain of PB1-F2 peptides delivered intranasally and identified residues L62, R75, R79 and L82 as essential for the induced lung immunopathology [48]. Additionally, these residues have been associated with enhanced pathogenicity in the context of bacterial secondary infection [49]. Three of these four residues are conserved in the alignment between PB1-F2 and IKKα (Fig. 2A). This region of similarity between PB1-F2 overlaps with part of the ubiquitin like domain (ULD) of IKKα. The ULD of IKKβ has been implicated in the dissociation of the kinase from p65 and it is suggested that the association between IKKβ and p65 may prevent the binding of p65 to its target DNA [50]. However, we were unable to demonstrate an increase in IKKβ-p65 interaction in cells expressing PB1-F2 (data not shown).

Phosphorylation of p65 by IKKβ at Ser468 [36] promotes its ubiquitination and degradation when bound to specific gene targets [37], [38], thus playing an important role in the control of NF-κB responses [51]. It was considered that PB1-F2 might influence p65 ubiquitination by favoring the IKKβ-mediated phosphorylation at Ser468 and induce p65 degradation. We found no evidence for increased ubiquitination of p65 in cells expressing PB1-F2 (data not shown). In this context, p65 ubiquitination occurs upon binding to κB sites [38], [51] and here we have shown that PB1-F2 is able to inhibit the binding of NF-κB to DNA.

PB1-F2 modulation of IKKβ activity in the nucleus may account for our observed inhibition of NF-κB signalling in transfected cells. Experiments using sodium pervanadate, which induces IKK-independent IκBα phosphorylation and degradation, showed that NF-κB DNA binding activity is significantly reduced in IKKβ^−/−^ cells [52]. This observation is remarkably similar to the inhibition of NF-κB binding to DNA observed in PB1-F2 expressing cells and also suggests that IKKβ may contribute to NF-κB signalling independently of its kinase activity. However the exact mechanism(s) by which IKKβ controls the expression of NF-κB dependent genes in the nucleus remains to be elucidated [52]. The ability of PB1-F2 proteins to modulate NF-κB signalling pathways did not correlate to their ability to interact with IKKβ, suggesting that PB1-F2 may recruit other protein(s) to repress IKKβ, and this may be differently regulated by the diverse PB1-F2 tested.

The observation that PB1-F2 Q/HK97 is able to inhibit NF-κB dependent transcription in cells overexpressing p65 raises the possibility that PB1-F2 from this virus might have a direct impact on this transcription factor, independent of its interaction with IKKβ. However, we found no evidence for direct interaction between p65 and PB1-F2 by immunoprecipitation (data not shown).

PB1-F2 has also been shown to interact with MAVS [29] and this interaction is expected to inhibit NF-κB activation induced by RIG-I and melanoma differentiation associated protein 5 (MDA-5) engagement. Therefore PB1-F2 may impact at different levels of NF-κB signalling which is reminiscent of the ability of NS1 to modulate different steps of the type I IFN induction pathway [10].

The results of deletions of PB1-F2 in the virus genome are conflicting. Goffic *et al* reported that deletion of PB1-F2 decreased NF-κB activation and subsequent reduction of IFNβ expression in A549 infected cells [30] and *in vivo* [31]. In contrast, similar experiments carried out by Dudek *et al*. showed increased IFNβ expression [27]. Conenello *et al.* [26] and Varga *et al*. [29] reported that PB1-F2 carrying a N66S substitution resulted in inhibition of IFN response both *in vivo* and *in vitro*. The reasons for these conflicting results may be associated with differences in the viruses used related to the level of N40 expression [47], [53]; differences in interaction with other virus proteins such as PB1 [24], [25], possibly to control the levels of PB1-F2 free in the cell to interact with host proteins such as IKKβ; and variability in the ability of PB1-F2 proteins to modify the host signalling cascades, as we observe here.

It was recently reported that NS1 is also able to interact with IKKβ, impairing IκBα phosphorylation and consequent translocation of NF-κB dimers to the nucleus [54]. It is remarkable that a virus has evolved two different proteins to interact with the same host kinase. This not only highlights the importance of regulating NF-κB signalling during influenza virus infection, but also raises several questions. Are these proteins able to interact with IKKβ at the same time and form a complex? Do they synergize with each other? Do they interact with IKKβ at different times post infection and, if so, do they compete for the same binding site on IKKβ? The answers to these questions are beyond the scope of this report. However it is worth noting that the possible competition for the same binding site on IKKβ would help to explain the conflicting results obtained with the PB1-F2 deletion mutants [26]–[31].

Preliminary data obtained in our laboratory indicated that deletion of the PB1-F2 ORF by mutation of the ATG and introduction of two STOP codons as described previously [22] did not increase NF-κB activation in influenza infected cells. However, the mutation of the PB1-F2 start codon has been shown to up-regulate N40 levels [47]. In light of this and the role of NS1 interacting with IKKβ, a mutant virus containing only the two stop codons and a deleted NS1 should be tested to unravel the potential role of the interaction between PB1-F2 and IKKβ in virus infection.

In summary, in this report we show that influenza virus protein PB1-F2 is able to modulate NF-κB signalling and interact with IKKβ. PB1-F2 inhibits NF-κB binding to DNA probably by impacting on IKKβ activity in the nucleus through a yet unknown mechanism(s). Given the key role of NF-κB signalling in the initiation and resolution of inflammation, this observation may shed light on the mechanisms by which PB1-F2 might be able to induce immunopathology.

## Supporting Information

Figure S1 **Alignment of the PB1-F2 protein sequences tested in this study.** Alignment of the PB1-F27 protein sequences using ClustalW (*denotes identical residues, : denotes conservative substitutions and denotes semi-conservative substitutions).
(TIF)

Figure S2 **PB1-F2 does not have a general impact on cell signaling.** A) Vero cells were co-transfected with a luciferase reporter plasmid containing six tandem copies of the IRF-1 gamma activated site (GAS) and either each of the different PB1-F2 expression vectors or the empty pcDNA4 plasmid along with the β-galactosidase control plasmid. Forty eight hours post-transfection, the cells were either induced or not induced with 1000 U ml^−1^ IFNγ for five hours, cell extracts were made and the luciferase and galactosidase activites were measured. B) Vero cells were co-transfected with a luciferase reporter plasmid containing four tandem copies of the 9–27 IFN-stimulated response element (ISRE) and either each of the different PB1-F2 expression vectors or the empty pcDNA4 plasmid along with the β-galactosidase control plasmid. Forty eight hours post-transfection, the cells were either induced or not induced with 1000 U ml^−1^ IFNα for five hours, cell extracts were made and the luciferase and galactosidase activites were measured. Data are representative of at least 3 independent experiments. Bars represent average values and standard deviations of firefly luciferase activities from triplicate samples normalized to the expression of galactosidase. No statistically significant differences from control empty vector were observed (one-way ANOVA followed by Dunnett's test).
(TIF)

Figure S3 **Expression of PB1-F2 constructs in transfected Vero cells.** Vero cells in a 24 well plate, were transfected with 600 ng of each of the different PB1-F2 constructs and PB1-F2 expression was detected by immunofluorescence using a monoclonal mouse anti-myc antibody (Sigma, M4439).
(TIF)

## References

[1] Medina RA, Garcia-Sastre A (2011) Influenza A viruses: new research developments. Nat Rev Microbiol 9: 590–603. 21747392 10.1038/nrmicro2613 PMC10433403

[2] Maines TR, Szretter KJ, Perrone L, Belser JA, Bright RA, et al (2008) Pathogenesis of emerging avian influenza viruses in mammals and the host innate immune response. Immunol Rev 225: 68–84. 18837776 10.1111/j.1600-065X.2008.00690.x

[3] Tumpey TM, Garcia-Sastre A, Taubenberger JK, Palese P, Swayne DE, et al (2005) Pathogenicity of influenza viruses with genes from the 1918 pandemic virus: functional roles of alveolar macrophages and neutrophils in limiting virus replication and mortality in mice. J Virol 79: 14933–14944. 16282492 10.1128/JVI.79.23.14933-14944.2005 PMC1287592

[4] Perrone LA, Plowden JK, Garcia-Sastre A, Katz JM, Tumpey TM (2008) H5N1 and 1918 pandemic influenza virus infection results in early and excessive infiltration of macrophages and neutrophils in the lungs of mice. PLoS Pathog 4: e1000115. 18670648 10.1371/journal.ppat.1000115 PMC2483250

[5] de Jong MD, Simmons CP, Thanh TT, Hien VM, Smith GJ, et al (2006) Fatal outcome of human influenza A (H5N1) is associated with high viral load and hypercytokinemia. Nat Med 12: 1203–1207. 16964257 10.1038/nm1477 PMC4333202

[6] Kobasa D, Jones SM, Shinya K, Kash JC, Copps J, et al (2007) Aberrant innate immune response in lethal infection of macaques with the 1918 influenza virus. Nature 445: 319–323. 17230189 10.1038/nature05495

[7] Baskin CR, Bielefeldt-Ohmann H, Tumpey TM, Sabourin PJ, Long JP, et al (2009) Early and sustained innate immune response defines pathology and death in nonhuman primates infected by highly pathogenic influenza virus. Proc Natl Acad Sci U S A 106: 3455–3460. 19218453 10.1073/pnas.0813234106 PMC2642661

[8] Medzhitov R, Horng T (2009) Transcriptional control of the inflammatory response. Nat Rev Immunol 9: 692–703. 19859064 10.1038/nri2634

[9] Hayden MS, Ghosh S (2008) Shared principles in NF-kappaB signaling. Cell 132: 344–362. 18267068 10.1016/j.cell.2008.01.020

[10] Hale BG, Randall RE, Ortin J, Jackson D (2008) The multifunctional NS1 protein of influenza A viruses. J Gen Virol 89: 2359–2376. 18796704 10.1099/vir.0.2008/004606-0

[11] Iwai A, Shiozaki T, Kawai T, Akira S, Kawaoka Y, et al (2010) Influenza A virus polymerase inhibits type I interferon induction by binding to interferon beta promoter stimulator 1. J Biol Chem 285: 32064–32074. 20699220 10.1074/jbc.M110.112458 PMC2952208

[12] Graef KM, Vreede FT, Lau YF, McCall AW, Carr SM, et al (2010) The PB2 subunit of the influenza virus RNA polymerase affects virulence by interacting with the mitochondrial antiviral signaling protein and inhibiting expression of beta interferon. J Virol 84: 8433–8445. 20538852 10.1128/JVI.00879-10 PMC2919034

[13] Chen W, Calvo PA, Malide D, Gibbs J, Schubert U, et al (2001) A novel influenza A virus mitochondrial protein that induces cell death. Nat Med 7: 1306–1312. 11726970 10.1038/nm1201-1306

[14] Jagger BW, Wise HM, Kash JC, Walters KA, Wills NM, et al (2012) An overlapping protein-coding region in influenza A virus segment 3 modulates the host response. Science 337: 199–204. 22745253 10.1126/science.1222213 PMC3552242

[15] McAuley JL, Hornung F, Boyd KL, Smith AM, McKeon R, et al (2007) Expression of the 1918 influenza A virus PB1-F2 enhances the pathogenesis of viral and secondary bacterial pneumonia. Cell Host Microbe 2: 240–249. 18005742 10.1016/j.chom.2007.09.001 PMC2083255

[16] McAuley JL, Chipuk JE, Boyd KL, Van De Velde N, Green DR, et al (2010) PB1-F2 proteins from H5N1 and 20 century pandemic influenza viruses cause immunopathology. PLoS Pathog 6: e1001014. 20661425 10.1371/journal.ppat.1001014 PMC2908617

[17] Gibbs JS, Malide D, Hornung F, Bennink JR, Yewdell JW (2003) The influenza A virus PB1-F2 protein targets the inner mitochondrial membrane via a predicted basic amphipathic helix that disrupts mitochondrial function. J Virol 77: 7214–7224. 12805420 10.1128/JVI.77.13.7214-7224.2003 PMC164823

[18] Zamarin D, Garcia-Sastre A, Xiao X, Wang R, Palese P (2005) Influenza virus PB1-F2 protein induces cell death through mitochondrial ANT3 and VDAC1. PLoS Pathog 1: e4. 16201016 10.1371/journal.ppat.0010004 PMC1238739

[19] Chanturiya AN, Basanez G, Schubert U, Henklein P, Yewdell JW, et al (2004) PB1-F2, an influenza A virus-encoded proapoptotic mitochondrial protein, creates variably sized pores in planar lipid membranes. J Virol 78: 6304–6312. 15163724 10.1128/JVI.78.12.6304-6312.2004 PMC416516

[20] Henkel M, Mitzner D, Henklein P, Meyer-Almes FJ, Moroni A, et al (2010) The proapoptotic influenza A virus protein PB1-F2 forms a nonselective ion channel. PLoS One 5: e11112. 20559552 10.1371/journal.pone.0011112 PMC2886074

[21] Mitzner D, Dudek SE, Studtrucker N, Anhlan D, Mazur I, et al (2009) Phosphorylation of the influenza A virus protein PB1-F2 by PKC is crucial for apoptosis promoting functions in monocytes. Cell Microbiol 11: 1502–1516. 19523156 10.1111/j.1462-5822.2009.01343.x

[22] Zamarin D, Ortigoza MB, Palese P (2006) Influenza A virus PB1-F2 protein contributes to viral pathogenesis in mice. Journal of Virology 80: 7976–7983. 16873254 10.1128/JVI.00415-06 PMC1563817

[23] Chen CJ, Chen GW, Wang CH, Huang CH, Wang YC, et al (2010) Differential localization and function of PB1-F2 derived from different strains of influenza A virus. J Virol 84: 10051–10062. 20660199 10.1128/JVI.00592-10 PMC2937816

[24] Mazur I, Anhlan D, Mitzner D, Wixler L, Schubert U, et al (2008) The proapoptotic influenza A virus protein PB1-F2 regulates viral polymerase activity by interaction with the PB1 protein. Cell Microbiol 10: 1140–1152. 18182088 10.1111/j.1462-5822.2008.01116.x

[25] McAuley JL, Zhang K, McCullers JA (2010) The effects of influenza A virus PB1-F2 protein on polymerase activity are strain specific and do not impact pathogenesis. J Virol 84: 558–564. 19828614 10.1128/JVI.01785-09 PMC2798424

[26] Conenello GM, Tisoncik JR, Rosenzweig E, Varga ZT, Palese P, et al (2011) A single N66S mutation in the PB1-F2 protein of influenza A virus increases virulence by inhibiting the early interferon response in vivo. J Virol 85: 652–662. 21084483 10.1128/JVI.01987-10 PMC3020033

[27] Dudek SE, Wixler L, Nordhoff C, Nordmann A, Anhlan D, et al (2011) The influenza virus PB1-F2 protein has interferon antagonistic activity. Biol Chem 392: 1135–1144. 22050228 10.1515/BC.2011.174

[28] Varga ZT, Ramos I, Hai R, Schmolke M, Garcia-Sastre A, et al (2011) The influenza virus protein PB1-F2 inhibits the induction of type I interferon at the level of the MAVS adaptor protein. PLoS Pathog 7: e1002067. 21695240 10.1371/journal.ppat.1002067 PMC3111539

[29] Varga ZT, Grant A, Manicassamy B, Palese P (2012) Influenza virus protein PB1-F2 inhibits the induction of type I interferon by binding to MAVS and decreasing mitochondrial membrane potential. J Virol 86: 8359–8366. 22674996 10.1128/JVI.01122-12 PMC3421771

[30] Le Goffic R, Bouguyon E, Chevalier C, Vidic J, Da Costa B, et al (2010) Influenza A virus protein PB1-F2 exacerbates IFN-beta expression of human respiratory epithelial cells. J Immunol 185: 4812–4823. 20844191 10.4049/jimmunol.0903952

[31] Le Goffic R, Leymarie O, Chevalier C, Rebours E, Da Costa B, et al (2011) Transcriptomic analysis of host immune and cell death responses associated with the influenza A virus PB1-F2 protein. PLoS Pathog 7: e1002202. 21901097 10.1371/journal.ppat.1002202 PMC3161975

[32] Visvanathan KV, Goodbourn S (1989) Double-stranded RNA activates binding of NF-kappa B to an inducible element in the human beta-interferon promoter. EMBO J 8: 1129–1138. 2663471 10.1002/j.1460-2075.1989.tb03483.x PMC400924

[33] Zell R, Krumbholz A, Eitner A, Krieg R, Halbhuber KJ, et al (2007) Prevalence of PB1-F2 of influenza A viruses. J Gen Virol 88: 536–546. 17251572 10.1099/vir.0.82378-0

[34] Sakurai H, Chiba H, Miyoshi H, Sugita T, Toriumi W (1999) IkappaB kinases phosphorylate NF-kappaB p65 subunit on serine 536 in the transactivation domain. J Biol Chem 274: 30353–30356. 10521409 10.1074/jbc.274.43.30353

[35] Yang F, Tang E, Guan K, Wang CY (2003) IKK beta plays an essential role in the phosphorylation of RelA/p65 on serine 536 induced by lipopolysaccharide. J Immunol 170: 5630–5635. 12759443 10.4049/jimmunol.170.11.5630

[36] Schwabe RF, Sakurai H (2005) IKKbeta phosphorylates p65 at S468 in transactivaton domain 2. FASEB J 19: 1758–1760. 16046471 10.1096/fj.05-3736fje

[37] Geng H, Wittwer T, Dittrich-Breiholz O, Kracht M, Schmitz ML (2009) Phosphorylation of NF-kappaB p65 at Ser468 controls its COMMD1-dependent ubiquitination and target gene-specific proteasomal elimination. EMBO Rep 10: 381–386. 19270718 10.1038/embor.2009.10 PMC2672889

[38] Mao X, Gluck N, Li D, Maine GN, Li H, et al (2009) GCN5 is a required cofactor for a ubiquitin ligase that targets NF-kappaB/RelA. Genes Dev 23: 849–861. 19339690 10.1101/gad.1748409 PMC2666342

[39] Pugin J, Schurer-Maly CC, Leturcq D, Moriarty A, Ulevitch RJ, et al (1993) Lipopolysaccharide activation of human endothelial and epithelial cells is mediated by lipopolysaccharide-binding protein and soluble CD14. Proc Natl Acad Sci U S A 90: 2744–2748. 7681988 10.1073/pnas.90.7.2744 PMC46172

[40] Rahman MM, McFadden G (2011) Modulation of NF-kappaB signalling by microbial pathogens. Nat Rev Microbiol 9: 291–306. 21383764 10.1038/nrmicro2539 PMC3611960

[41] Schmolke M, Manicassamy B, Pena L, Sutton T, Hai R, et al (2011) Differential contribution of PB1-F2 to the virulence of highly pathogenic H5N1 influenza A virus in mammalian and avian species. PLoS Pathog 7: e1002186. 21852950 10.1371/journal.ppat.1002186 PMC3154844

[42] Meunier I, von Messling V (2012) PB1-F2 modulates early host responses but does not affect the pathogenesis of H1N1 seasonal influenza virus. J Virol 86: 4271–4278. 22318139 10.1128/JVI.07243-11 PMC3318652

[43] Sun K, Metzger DW (2008) Inhibition of pulmonary antibacterial defense by interferon-gamma during recovery from influenza infection. Nat Med 14: 558–564. 18438414 10.1038/nm1765

[44] Fong CH, Bebien M, Didierlaurent A, Nebauer R, Hussell T, et al (2008) An antiinflammatory role for IKKbeta through the inhibition of “classical” macrophage activation. J Exp Med 205: 1269–1276. 18490491 10.1084/jem.20080124 PMC2413025

[45] Park JM, Greten FR, Wong A, Westrick RJ, Arthur JS, et al (2005) Signaling pathways and genes that inhibit pathogen-induced macrophage apoptosis–CREB and NF-kappaB as key regulators. Immunity 23: 319–329. 16169504 10.1016/j.immuni.2005.08.010

[46] Li ZW, Chu W, Hu Y, Delhase M, Deerinck T, et al (1999) The IKKbeta subunit of IkappaB kinase (IKK) is essential for nuclear factor kappaB activation and prevention of apoptosis. J Exp Med 189: 1839–1845. 10359587 10.1084/jem.189.11.1839 PMC2193082

[47] Wise HM, Foeglein A, Sun J, Dalton RM, Patel S, et al (2009) A complicated message: Identification of a novel PB1-related protein translated from influenza A virus segment 2 mRNA. J Virol 83: 8021–8031. 19494001 10.1128/JVI.00826-09 PMC2715786

[48] Alymova IV, Green AM, van de Velde N, McAuley JL, Boyd KL, et al (2011) Immunopathogenic and antibacterial effects of H3N2 influenza A virus PB1-F2 map to amino acid residues 62, 75, 79, and 82. J Virol 85: 12324–12333. 21937639 10.1128/JVI.05872-11 PMC3209399

[49] Weeks-Gorospe JN, Hurtig HR, Iverson AR, Schuneman MJ, Webby RJ, et al (2012) Naturally occurring swine influenza A virus PB1-F2 phenotypes that contribute to superinfection with Gram-positive respiratory pathogens. J Virol 86: 9035–9043. 22674997 10.1128/JVI.00369-12 PMC3416121

[50] May MJ, Larsen SE, Shim JH, Madge LA, Ghosh S (2004) A novel ubiquitin-like domain in IkappaB kinase beta is required for functional activity of the kinase. J Biol Chem 279: 45528–45539. 15319427 10.1074/jbc.M408579200

[51] Saccani S, Marazzi I, Beg AA, Natoli G (2004) Degradation of promoter-bound p65/RelA is essential for the prompt termination of the nuclear factor kappaB response. J Exp Med 200: 107–113. 15226358 10.1084/jem.20040196 PMC2213320

[52] Gloire G, Horion J, El Mjiyad N, Bex F, Chariot A, et al (2007) Promoter-dependent effect of IKKalpha on NF-kappaB/p65 DNA binding. J Biol Chem 282: 21308–21318. 17537731 10.1074/jbc.M610728200

[53] Tauber S, Ligertwood Y, Quigg-Nicol M, Dutia BM, Elliott RM (2012) Behaviour of influenza A viruses differentially expressing segment 2 gene products in vitro and in vivo. J Gen Virol 93: 840–849. 22190016 10.1099/vir.0.039966-0

[54] Gao S, Song L, Li J, Zhang Z, Peng H, et al (2012) Influenza A virus-encoded NS1 virulence factor protein inhibits innate immune response by targeting IKK. Cell Microbiol 14: 1849–1866. 22891964 10.1111/cmi.12005

