# Supplementary figures and images for: The Influenza Virus Protein PB1-F2 Interacts with IKKβ and Modulates NF-κB Signalling
Source: PLoS One. 2013 May 21;8(5):e63852. doi: 10.1371/journal.pone.0063852 (PMC3660569; doi:10.1371/journal.pone.0063852)

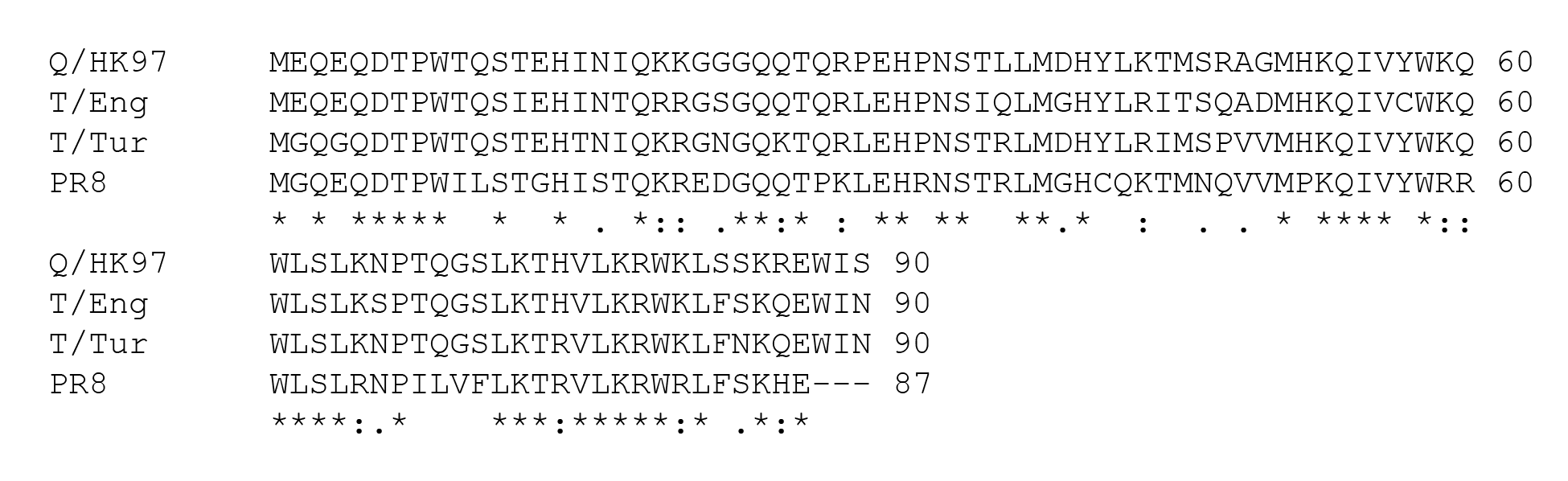

Supplement: Figure S1 — Alignment of the PB1-F2 protein sequences tested in this study. Alignment of the PB1-F27 protein sequences using ClustalW (*denotes identical residues, : denotes conservative substitutions and denotes semi-conservative substitutions). (TIF) [file pone.0063852.s001.tif]

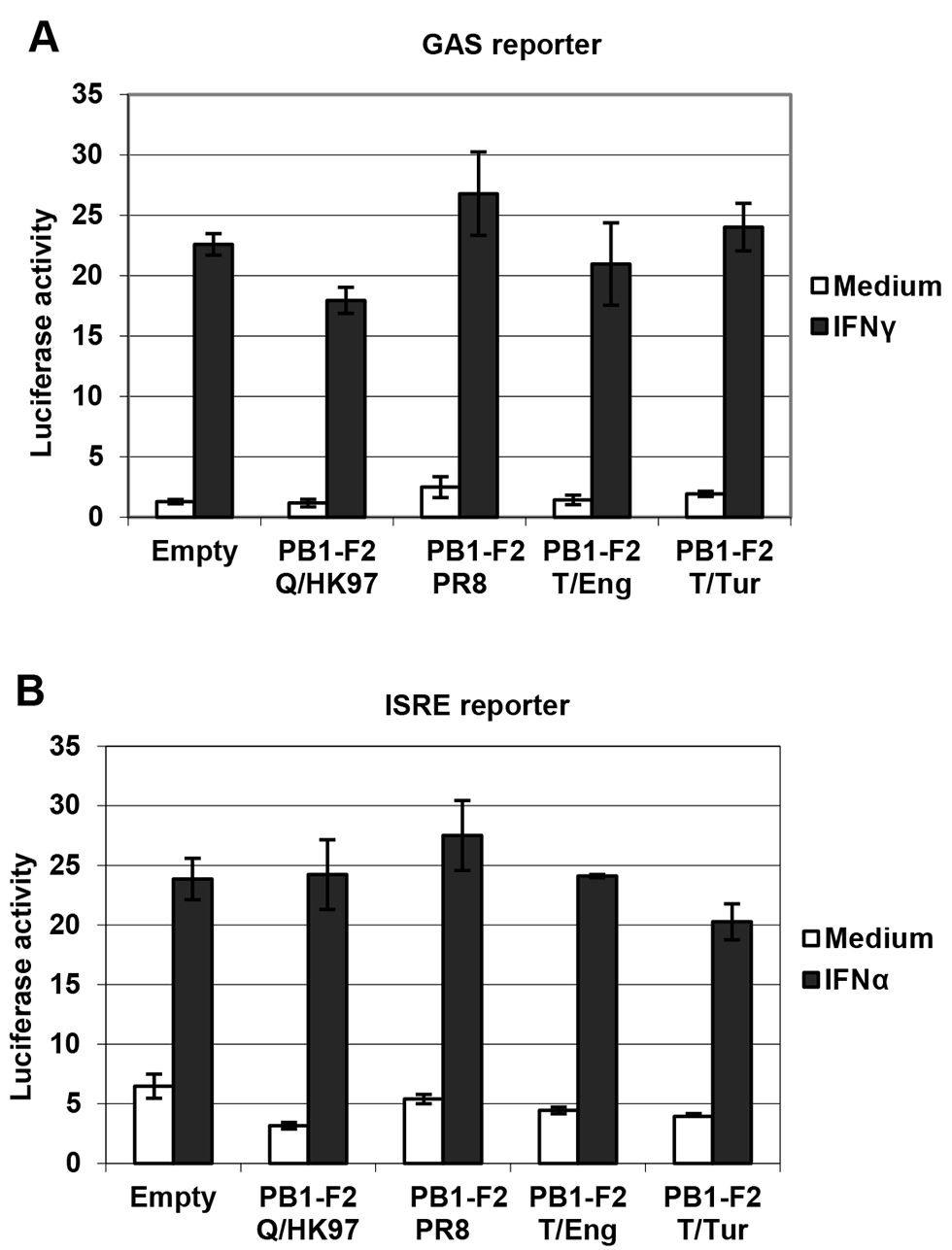

Supplement: Figure S2 — PB1-F2 does not have a general impact on cell signaling. A) Vero cells were co-transfected with a luciferase reporter plasmid containing six tandem copies of the IRF-1 gamma activated site (GAS) and either each of the different PB1-F2 expression vectors or the empty pcDNA4 plasmid along with the β-galactosidase control plasmid. Forty eight hours post-transfection, the cells were either induced or not induced with 1000 U ml−1 IFNγ for five hours, cell extracts were made and the luciferase and galactosidase activites were measured. B) Vero cells were co-transfected with a luciferase reporter plasmid containing four tandem copies of the 9–27 IFN-stimulated response element (ISRE) and either each of the different PB1-F2 expression vectors or the empty pcDNA4 plasmid along with the β-galactosidase control plasmid. Forty eight hours post-transfection, the cells were either induced or not induced with 1000 U ml−1 IFNα for five hours, cell extracts were made and the luciferase and galactosidase activites were measured. Data are representative of at least 3 independent experiments. Bars represent average values and standard deviations of firefly luciferase activities from triplicate samples normalized to the expression of galactosidase. No statistically significant differences from control empty vector were observed (one-way ANOVA followed by Dunnett's test). (TIF) [file pone.0063852.s002.tif]

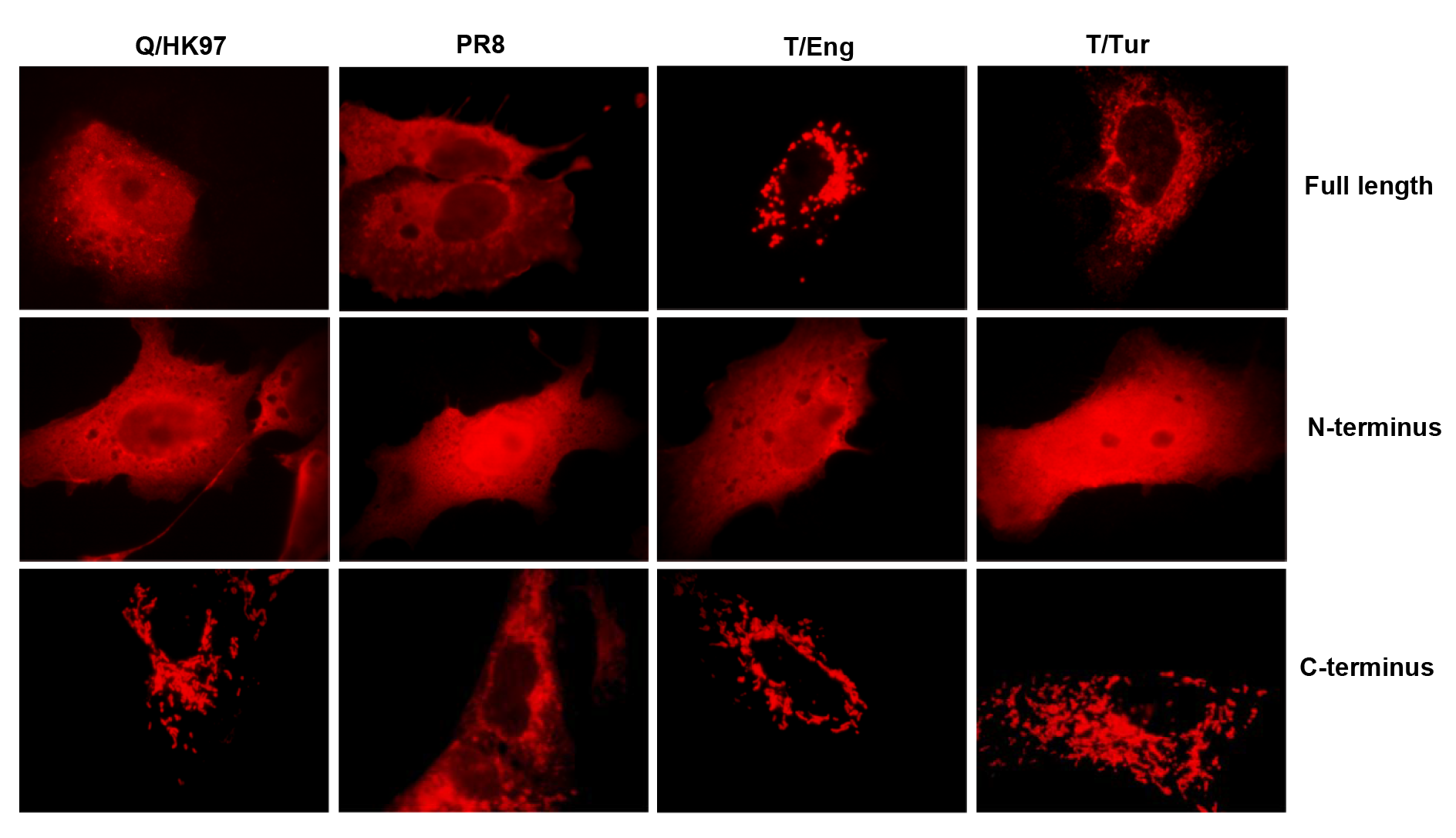

Supplement: Figure S3 — Expression of PB1-F2 constructs in transfected Vero cells. Vero cells in a 24 well plate, were transfected with 600 ng of each of the different PB1-F2 constructs and PB1-F2 expression was detected by immunofluorescence using a monoclonal mouse anti-myc antibody (Sigma, M4439). (TIF) [file pone.0063852.s003.tif]
